# Effects of a ketogenic diet in overweight women with polycystic ovary syndrome

**DOI:** 10.1186/s12967-020-02277-0

**Published:** 2020-02-27

**Authors:** Antonio Paoli, Laura Mancin, Maria Cristina Giacona, Antonino Bianco, Massimiliano Caprio

**Affiliations:** 1 Department of Biomedical Sciences, University of Padua, Padua, Italy; 2 Research Center for High Performance Sport, UCAM, Catholic University of Murcia, Murcia, Spain; 3 Human Inspired Technology Research Center HIT, University of Padua, Padua, Italy; 4 Department of Neuroscience, Biomedicine and Movement Sciences, University of Verona, Verona, Italy; 5 Department of Psychology, Educational Science and Human Movement, University of Palermo, Palermo, Italy; 6 Laboratory of Cardiovascular Endocrinology, IRCCS San Raffaele Pisana, Rome, Italy; 7 Department of Human Sciences and Promotion of the Quality of Life, San Raffaele Roma Open University, Rome, Italy

**Keywords:** Overweight, Ketogenic diet, PCOS, Hyperinsulinemia, LCKD, Ketone bodies, Low carbohydrate diet

## Abstract

**Background:**

Polycystic ovary syndrome (PCOS) is the most common endocrine disorder in women during reproductive age. It is characterised clinically by oligo-ovulation or anovulation, hyper-androgenism, and the presence of polycystic ovaries. It is associated with an increased prevalence of metabolic syndrome, cardiovascular disease and type 2 diabetes. The onset of PCOS has been associated to several hereditary and environmental factors, but insulin resistance plays a key pathogenetic role. We sought to investigate the effects of a ketogenic diet (KD) on women of childbearing age with a diagnosis of PCOS.

**Methods:**

Fourteen overweight women with diagnosis of PCOS underwent to a ketogenic Mediterranean diet with phyoextracts (KEMEPHY) for 12 week. Changes in body weight, body mass index (BMI), fat body mass (FBM), lean body mass (LBM), visceral adipose tissue (VAT), insulin, glucose, HOMA-IR, total cholesterol, low density lipoprotein (LDL), high density lipoprotein (HDL), triglycerides (TGs), total and free testosterone, luteinizing hormone (LH), follicle stimulating hormone (FSH); dehydroepiandrosterone sulfate (DHEAs), estradiol, progesterone, sex hormone binding globulin (SHBG) and Ferriman Gallwey score were evaluated.

**Results:**

After 12 weeks, anthropometric and body composition measurements revealed a significant reduction of body weight (− 9.43 kg), BMI (− 3.35), FBM (8.29 kg) and VAT. There was a significant, slightly decrease of LBM. A significant decrease in glucose and insulin blood levels were observed, together with a significant improvement of HOMA-IR. A significant decrease of triglycerides, total cholesterol and LDL were observed along with a rise in HDL levels. The LH/FSH ratio, LH total and free testosterone, and DHEAS blood levels were also significantly reduced. Estradiol, progesterone and SHBG increased. The Ferriman Gallwey Score was slightly, although not significantly, reduced.

**Conclusions:**

Our results suggest that a KD may be considered as a valuable non pharmacological treatment for PCOS. Longer treatment periods should be tested to verify the effect of a KD on the dermatological aspects of PCOS.

*Trial registration* Clinicaltrial.gov, NCT04163120, registrered 10 November 2019, retrospectively registered, https://clinicaltrials.gov.

## Background

Polycystic ovarian syndrome (PCOS) is considered the most common endocrine disorder in women in the reproductive age, with an estimated prevalence ranging from 6 to 15%, depending on the diagnostic criteria used. PCOS, in fact, is an heterogeneous condition with variable phenotypic expression leading to significant controversy on the diagnostic criteria [1].

Women with PCOS often seek care for menstrual disturbances (oligomenorrhea, amenorrhea, prolonged irregular menstrual bleeding), clinical manifestations of hyperandrogenism and infertility. Hirsutism is a common clinical presentation of hyperandrogenism occurring in up to 70% of women with PCOS and is evaluated using the Ferriman–Gallwey scoring system [2].

Common signs of PCOS not included in diagnostic criteria are represented by insulin resistance, reversal of the FSH/LH ratio and obesity, which is an important clinical feature of PCOS. Women with PCOS have increased visceral and subcutaneous body fat due to higher androgen levels. Obesity also plays a significant role in explaining the metabolic characteristics of PCOS: patients display an atherogenic lipid profile, associated with elevated levels of low-density lipoprotein, triglycerides and cholesterol, along with reduced levels of high-density lipoprotein [3]. However, it is important to remark that these metabolic abnormalities may also be present in non-obese patients [4]. The positive correlations between hyperinsulinemia and androgen levels suggested that insulin contributes to hyperandrogenism in women with PCOS. The ovaries of PCOS patients usually maintain a normal response to insulin. A partial elucidation of this mechanism is explained by the action of insulin on the ovary through the IGF-1 receptor. This binding occurs when insulin reaches high concentrations, as in compensatory hyperinsulinemia. Insulin actions on the ovary are also mediated by the glycan molecules that contain D-chiro-inositol (DCI) [5], a different second messenger from the classical one activated by phosphorylation of the receptor at tyrosine level in other tissues. Hyperinsulinemia stimulates thecal cell proliferation, amplifies LH-mediated androgen secretion and increases expression of LH and IGF-1 receptor [6]. Furthermore, high insulin levels inhibit both the production of sex hormone binding globulin (SHBG) by the liver, causing increased levels of free testosterone [7], and the synthesis of IGF-BP1, increasing level of free IGF-1 [8].

Interestingly, excess carbohydrate intake and low-grade inflammation mutually interact with insulin resistance and hyperandrogenism to reinforce the metabolic phenotype of PCOS [9]. In fact, acute hyperglycaemia is known to increase inflammation and oxidative stress through generation of reactive oxygen species (ROS) [10]. PCOS women present a peculiar dietary pattern, characterised by reduced use of extra-virgin olive oil, legumes, seafood and nuts, a lower amount of complex carbohydrate, fiber, monounsaturated fatty acids, and higher simple carbohydrates, total fat and saturated fatty acid, compared to normal women. These nutritional habits are associated to an adverse body composition, characterised by reduced fat-free mass [11].

A univocal therapy for PCOS does not exist; the peculiar heterogeneity of this pathology requires that the treatment should be personalized, depending on the clinical presentation and needs of the patient.

The current guidelines as first-line treatment for menstrual irregularities, acne and hirsutism recommend hormonal contraceptives, at any age. Antiandrogens are suggested in the case that estroprogestinics are contraindicated or in the presence of severe hirsutism.

Metformin has long been used in therapeutic protocols, although alternatives are investigated, because of gastroenteric side effects; inositol represents an alternative approach. Anyway, metformin does not increase weight loss in patients treated with lifestyle modifications (diet and exercise programs). Therefore, diet and exercise, not metformin, should be the first line of therapy in obese women with PCOS. Metformin should be considered if the patient fails with diet and exercise [12]. Weight loss represents the most important factor to improve PCOS phenotype. A 5–10% weight loss improves ovulatory function and pregnancy rates, with reduction of insulin and free testosterone levels. However, even though lifestyle modification based on the principles of caloric restriction remains a primary therapy for PCOS and caloric restriction seems more important than macronutrient composition [13, 14], little data are available about diet’s macronutrient modification as therapeutic approach [15–17]. Indeed, it is controversial whether diet composition per se has an effect on reproductive and metabolic outcomes. Blood glucose levels are affected by carbohydrate intake and regulate insulin secretion from the pancreas, so very-low carbohydrates diets may be superior to standard hypocaloric diets in terms of improving fertility, endocrine/metabolic parameters, weight loss and satiety in women with PCOS [18]. Considering the all aforementioned conditions it would be reasonable that a ketogenic diet (KD) might has positive effects on PCOS. A KD is a nutritional protocol in which carbohydrates are lower than 30 g per day or 5% of total energy intake [19–21] relative increase in the proportions of protein and fat. The reduction of the amount of circulating glucose and insulin produces a reduction of the oxidation of glucose and an increase of the fat oxidation as showed by the reduction of the respiratory ratio [22] Another important effects of KD for PCOS is the activation of AMPK and SIRT-1, even in the absence of caloric deprivation [23]. Once activated, SIRT1 and AMPK produce beneficial effects on glucose homeostasis and improve insulin sensitivity [24].

The therapeutic role of KD has been investigated for a long time and several works have supported the thesis that physiological ketosis can be useful in many pathological conditions, such as epilepsy, neurological diseases, cancer (with a ketogenic isocaloric diet) [25] and obesity, type 2 diabetes, acne, and the amelioration of respiratory and cardiovascular disease risk factors (with a generally low calorie ketogenic diet) [26–28]. This is an important aim, since the use of food as a drug has very relevant social and economic implications, both in economic and social terms. In PCOS, evidence for the effects of KD are scarce: only a small uncontrolled pilot study [29] showed a significant reduction in body weight, free testosterone, LH to FSH ratio, and fasting insulin after a KD regimen, suggesting favourable effects in affected patients. Other data describe several mechanisms consistent with the favourable effects of such diet therapy [30–32]. A recent position statement of the Italian Society of Endocrinology suggested a weight-loss program with a very low calorie ketogenic diet for overweight/obese patients with PCOS) not responsive to multicomponent standardized diet to improve insulin resistance, ovulatory dysfunctions and hyperandrogenemia, even if further controlled studies are deemed necessary to confirm the beneficial effects of KD in this clinical context [28].

Thus, aim of the present study was to determine the effects of a ketogenic diet (KD) in women of childbearing age with a diagnosis of PCOS. We hypothesized that a modified KD (KEMEPHY diet) would lead to an improvement in body weight, plasma cholesterol, triglycerides, hyperinsulinemia and hormonal outcomes.

## Materials and methods

### Study design

This was a 12 weeks, single-arm study. The outcome measures were body weight, BMI, FBM, LBM, FBM percentage, LBM percentage, glucose, insulin, HOMA-IR, total cholesterol, HDL, LDL, triglycerides, total testosterone, free testosterone, progesterone, estradiol, LH, FSH, DHEAS, LH/FSH ratio, SHBG and Ferriman Gallwey Score. The study protocol complied with all tenets of the Helsinki declaration. All patients provided written informed consent before the beginning of the study. The study was approved by the ethical committee of the Department of Biomedical Sciences (HEC-DSB13/16). This trial was retrospectively registered on Clinicaltrial.gov, NCT04163120, registered 10 November 2019, https://clinicaltrials.gov.

### Participants

**Fig. 1:**
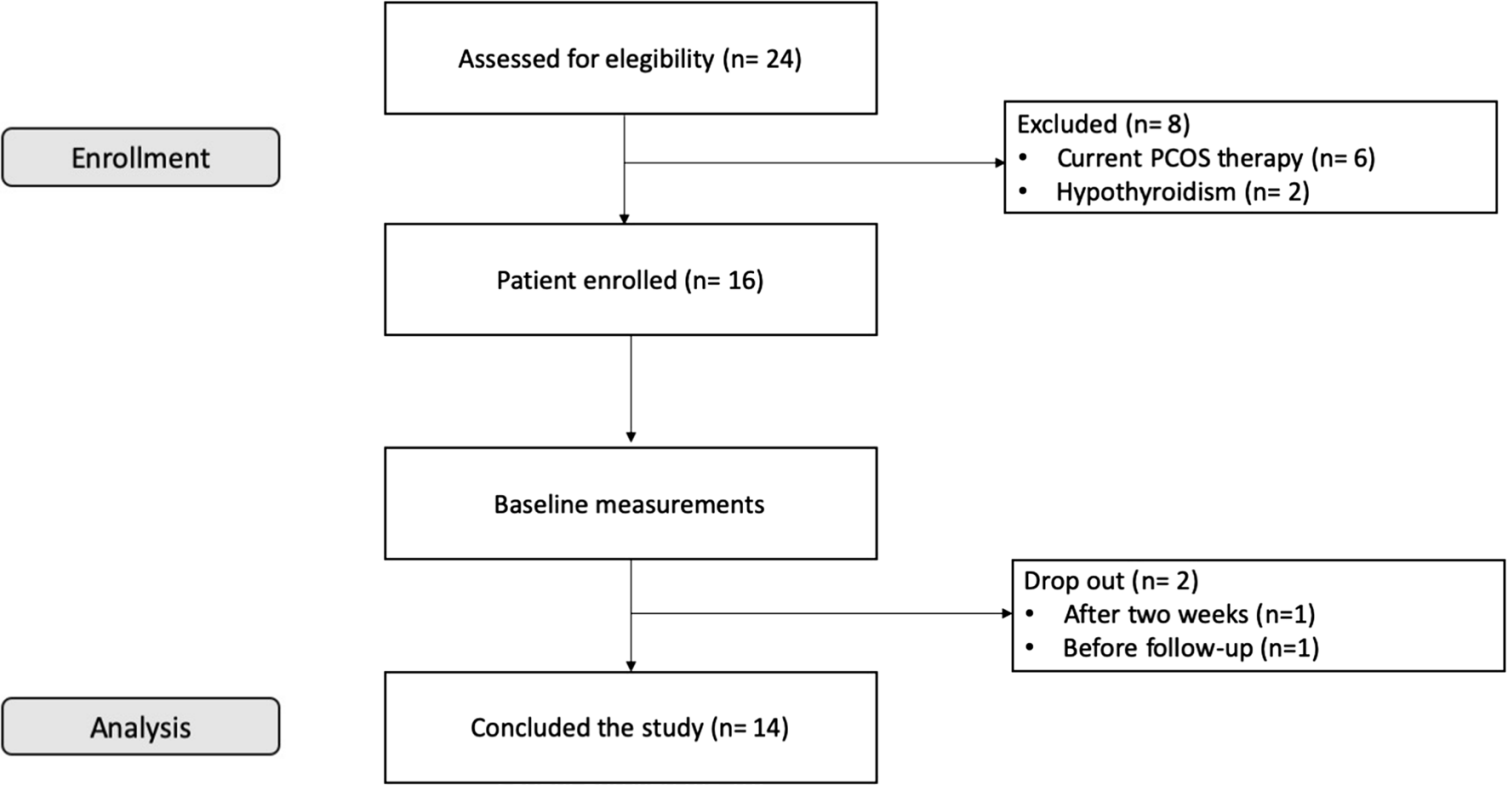
Flow chart of experimental design. Recruitment of patients. Further details are reported in the text

Twenty-four overweight women were enrolled trough public announcement in medical centers in Padova and Vicenza territory. Inclusion criteria were: diagnosis of PCOS according Rotterdam Criteria (at least 2 of 3 between oligo/amenorrhea or amenorrhea, clinical signs of hyperandrogenism and polycystic ovary confirmed with ultrasound [33]), fertile age (18–45 years); BMI ≥ 25 kg/m^2^, desire to lose weight; acceptance not to use contraceptives during the experimental period. Exclusion criteria were pregnancy and lactation, hormonal therapy and/or insulin-sensitizers in the last 2 months, hepatic, renal and heart diseases, local treatment for hirsutism, moreover, according to Rotterdam criteria, other aetiologies (congenital adrenal hyperplasia, androgen secreting tumours, Cushing syndrome) have been excluded. Six women were excluded for current PCOS pharmacological therapy and 2 for diagnosticated clinical hypothyroidism. One left the study after 2 weeks and one withdrew before the follow up; thus 14 subjects (age 27.5 ± 8.3 years; height 168 ± 4.3 cm; weight 81.19 ± 8.44 kg) concluded the study (Fig. 1).

Before starting the dietary protocol participants came to the Nutrition and Exercise Physiology Laboratory of the Department of Biomedical Sciences of the University of Padua for the basal measurements. Patients underwent a detailed anamnesis and physical examination, and plasma analysis for glucose, insulin, total cholesterol, HDL, LDL, triglycerides (TGs), total testosterone, free testosterone, progesterone, estradiol, LH, FSH, DHEAS and SHBG were carried out.

### Anthropometric measurements

Body weight was measured to the nearest 0.1 kg using an electronic scale (Tanita BWB-800 Medical Scales, USA), and height to the nearest 1 cm using a wall-mounted Harpenden portable stadiometer (Holtain Ltd, UK). Body mass index (BMI) was calculated in kg/m^2^. Waist circumference was measured as the smallest circumference between the lowest rib and the iliac crest [34, 35].

### Body composition analysis

Body composition (fat body mass FBM, lean body mass LBM and visceral adipose tissue VAT) was analysed by Dual X Ray Absorptiometry (DEXA) Hologic Horizon^TM^ QDR RSeries Bedford, Massachusetts, USA. Daily quality control scans were acquired during the study period. No hardware or software changes were made during the course of the trial. Subjects were scanned using standard positioning protocols, while wearing only light clothing. Regional analysis of the trunk and visceral adipose tissue VAT were assessed according to anatomical landmarks by the same technician using computer algorithms (Apex System).

### Blood exams

Blood samples were taken from antecubital vein and collected into BD Vacutainers Tubes (SST^TM^ II Advance, REF 367953). After blood sampling, samples were centrifuged (4000 RPM at 4 °C using centrifuge J6-MC by Beckman). The serum, so obtained, was aliquoted and stored at − 80 °C. All samples were analysed in the same analytical session for each test using the same reagent lot and having CV intraassay < 7%. Before the analytical session, the serum samples were thawed overnight at 4 °C and then mixed. Total testosterone, DHEAS, progesterone, estradiol, FSH, LH, insulin and SHBG were measured by immunochemiluminescent method (Roche Cobas e601, Roche Diagnostics, Mannheim, Germany), blood glucose by enzymatic method with esokinase (Roche Cobas e702, Roche Diagnostics, Mannheim, Germany), total cholesterol, HDL, and LDL by enzymatic colorimetric in homogenous phase (Roche Cobas e702, Roche Diagnostics, Mannheim, Germany), TGs by an enzymatic colorimetric method (Roche Cobas e702, Roche Diagnostics, Mannheim, Germany). Free testosterone was measured by RIA radioimmunological test (Beckman Coulter). The hormonal status evaluation was performed during the follicular phase of the menstrual cycle, between the 1st and the 7th day. HOMA-IR was calculated according to the formula “insulinemia (μU/mL) × glycemia (mmol/L)/22.5. At the end of the 12 weeks, the patients were re-evaluated in the same way and the collected clinical data and DEXA scan results data was statistically analyzed. Levels of serum 3-hydroxybutyrate (BHB), BHB is the most important indicator of ketosis i.e. blood level of ketone bodies (KBs), were assessed weekly using Precision Xtra^®^ Blood β-Ketone Test Strips and Precision Xtra^®^ [36] (Abbott Laboratories, Illinois 60064-3500, USA) which measures blood BHB level in fresh capillary whole blood from the fingertip between 0.0 and 8.0 mmol/L. The puncture was performed with the lancet Accu-Chek Softclix (Roche, Monza MB, Italy) on clean, dry and warm fingers. Hirsutism was considered positive when the Ferriman Gallwey Score was > 8.

### Diet

A modified KD protocol was used. The KEMEPHY diet [37–42] is a Mediterranean eucaloric ketogenic protocol (about 1600/1700 kcal/day) with the use of some phytoextracts. During this protocol subjects are allowed to eat with no limits green leafy vegetables, cruciferous, zucchini, cucumbers and eggplants. The quantity of meat, eggs and fish was limited (120 g of meat or 20 g of fish or 2 eggs) (see Table 1). Moreover, subjects daily consumed four food supplements and liquid herbal extracts. Food supplements are high proteins (19 g/portion) and very low carbohydrate (3.5 g/portion) formulas simulating the aspect and taste of common carbohydrate rich foods added with dry phytoextracts [43]. Liquid herbal extracts were used for their draining/toning activity, useful to reduce some commonly reported light side effects of ketogenic diets as constipation, headache and halitosis. Herbal extracts are reported in more details in our previous publication (Table 2) [37].

**Table 1:** KEMEPHY diet composition

	g	% Total E	Kcal
Daily total energy			1672 ± 90
Carbohydrate	20.3 ± 5.2	4.8 ± 1.2	81.5 ± 18.9
Fat	132.4 ± 11.7	71.1 ± 9.3	1188.2 ± 100.2
Protein	100.8 ± 8.6	24.1 ± 5.6	403.4 ± 45.3
Fats distribution		% total fats	
Saturated		38.8 ± 6.9	
Monounsaturated		50.9 ± 6.5	
Polyunsaturated		9.7 ± 5.6	
Protein (g/Kg body weight)	1.23 ± 0.8

**Table 2:** Plants extract composition

Plant extracts	Composition
Extracts 1, ml/day	Durvillea antarctica, black radish, mint, liquorice, artichoke, horsetail, burdock, dandelion, rhubarb, gentian, lemon balm, chinaroot, juniper, spear grass, elder, fucus, anise, parsley, bearberry, horehound
Extracts 2, ml/day	Horsetail, asparagus, birch, cypress, couch grass, corn, dandelion, grape, fennel, elder, rosehip, anise
Extracts 3, ml/day	Eleuthero, eurycoma longifolia, ginseng, corn, miura puama, grape, guaranà, arabic coffee, ginger
Extracts 4, ml/day	*Linum usitatissimum* L., *Gelidium amansii*, *Rheum officinalis* L., *Cynara scolymus* L., *Matricaria chamomilla* L., *Gentiana lutea* L., *Mentha piperita* L., *Pimpinella anisum* L., *Glycyrrhiza glabra* L., *Raphanus sativus* L., *Foeniculum vulgare* Mill., *Althaea officinalis* L., *Melissa officinalis* L., *Juniperus communis* L.

### Statistical analysis

A Student’s t test was used to compare parameters before and after 12 weeks of the KD, using the software package GraphPad Prism version 8.00 for Mac, GraphPad Software, San Diego California USA. All data are expressed as mean ± standard deviation. Normality of data was assessed through the D’Agostino Pearson test. Significance was considered at a value of p < 0.05.

## Results

**Fig. 2:**
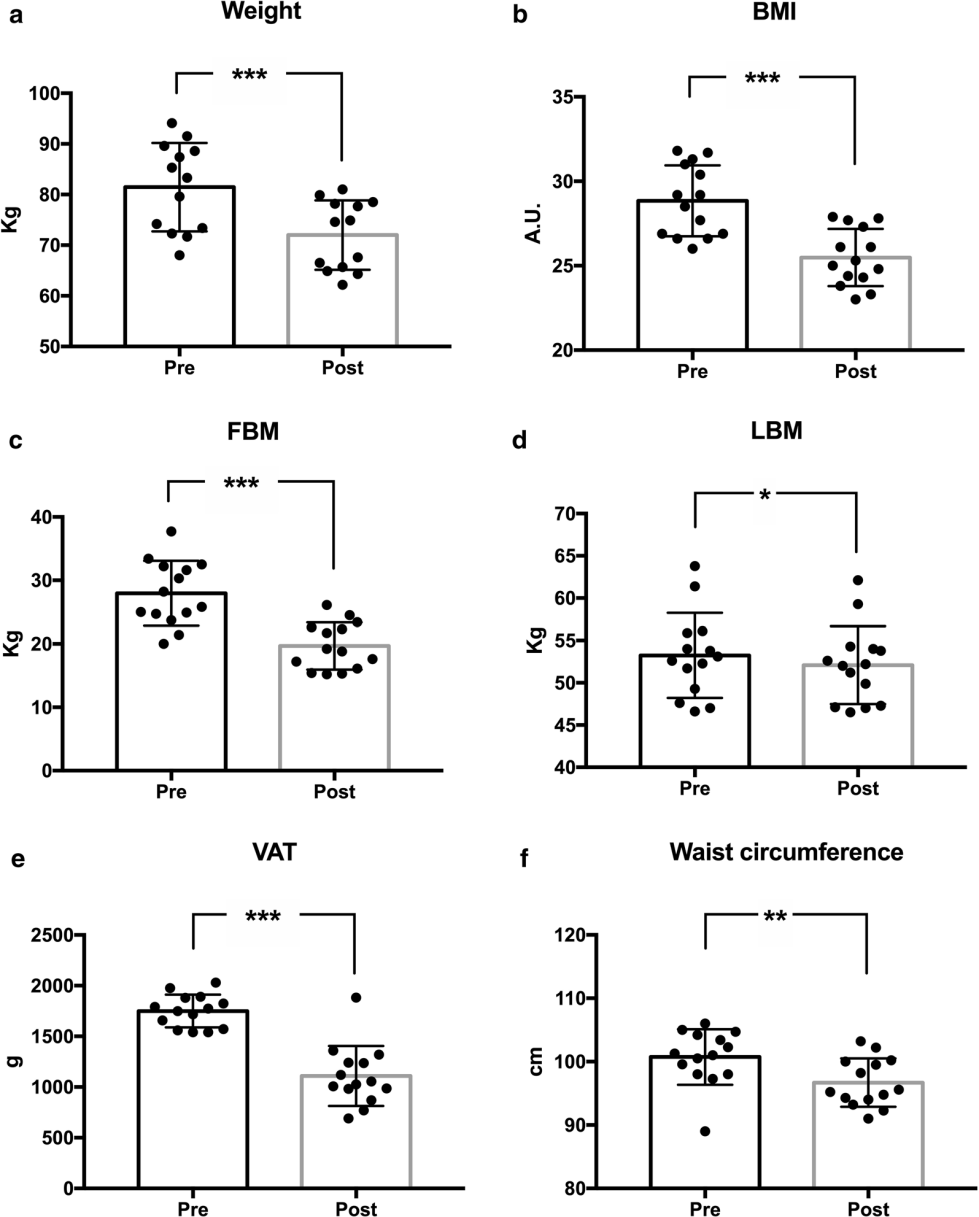
Changes in anthropometric variables after 12 weeks of KD. *BMI* body mass index, *FBM* fat body mass, *LBM* mean body mass. Standard deviation is represented in the figure. ***p < 0.0001; **p = 0.0015; *p = 0.0205

Anthropometric and body composition measurements revealed an average weight loss of 9.43 kg (pre 81.19 ± 8.44 kg vs post 71.76 ± 6.66 kg; p < 0.0001) and significant reductions (− 3.35) of BMI (pre 28.84 ± 2.10 vs post 25.49 ± 1.69; p < 0.0001) and of FBM (− 8.29 kg) (pre 27.96 ± 5.11 kg vs post 19.67 ± 3.72 kg; p < 0.0001). LBM absolute value showed a slightly significant decrease (pre 53.23 ± 5.02 kg vs post 52.09 ± 4.60 kg), but its percentage value was slightly increased (pre 65.74 ± 3.75% vs post 72.71 ± 3.55%; p < 0.0001), with an overall improvement in body composition. VAT showed a very significant (pre 1750 ± 181.58 grams vs. post 1110,36 ± 189.23; p < 0.0001) decrease and also waist circumference decreased in a significant manner (pre 100.7 ± 4.81 vs post 96.69 ± 3.82; p = 0.0015). Anthropometric and body composition results are presented as mean ± SD in Fig. 2.

### Metabolic biomarkers

Ketone bodies were measured every other day for the first 6 days, then every 6 days. The mean BHB value was 0.31 ± 0.18 mmol/L for the first 6 days and 1.77 ± 0.55 from day 7 to day 84. Patients at the beginning of the study had a HOMA-IR higher than 2.5, confirming insulin resistance. At the end of the study, a significant decrease was observed in glucose (pre 5.10 ± 0.25 mmol/L vs post 4.64 ± 0.24 mmol/L; p < 0.0001) and insulin (pre 12.62 ± 0.48 μU/mL vs post 11.31 ± 0.60 μU/mL; p < 0.0001) and consequently in the HOMA-IR (pre 2.85 ± 0.15 vs post 2.32 ± 0.13; p < 0.0001).There were significant changes in lipid profiles with reductions in triglycerides (pre 2.31 ± 0.40 mmol/L vs post 1.87 ± 0.27 mmol/L; p < 0.0008), total cholesterol (pre 5.36 ± 0.36 mmol/L vs post 4.72 ± 0.33 mmol/L; p < 0.0001) and LDL (pre 3.11 ± 0.60 mmol/L vs post 2.33 ± 0.17 mmol/L; p < 0.0001) along with a rise in HDL levels (pre 1.79 ± 0.41 mmol/L vs post 2.02 ± 0.43 mmol/L; p < 0.0001). The data are reported as mean ± SD in Table 3.

**Table 3:** Metabolic biomarkers before and after 12 weeks of KD

	Pre	Post	P value
Glucose (mmol/L)	5.10 ± 0.25	4.64 ± 0.24	< 0.0001
Insulin (μU/mL)	12.62 ± 0.48	11.31 ± 0.60	< 0.0001
HOMA-IR	2.85 ± 0.15	2.32 ± 0.13	< 0.0001
Triglycerides (mmol/L)	2.31 ± 0.40	1.87 ± 0.27	< 0.0008
Total cholesterol (mmol/L)	5.36 ± 0.36	4.72 ± 0.33	< 0.0001
LDL (mmol/L)	3.11 ± 0.60	2.33 ± 0.17	< 0.0001
HDL (mmol/L)	1.79 ± 0.41	2.02 ± 0.43	= 0.0146

Standard deviation is reported

*LDL* low density lipoprotein, *HDL* high density lipoprotein

### Endocrine biomarkers

The characteristic reversal of the LH/FSH ratio was observed at the beginning of the study and disappeared after 12 weeks (pre 2.00 ± 0.30 vs post 1.15 ± 0.20; p < 0.0001).

Compared to basal values, there was also a significant decrease in plasma concentrations of LH (pre 10.24 ± 1.43 vs post 6.41 ± 1.46; p < 0.0001), total testosterone (pre 47.43 ± 6.08 ng/dL vs post 40.71 ± 5.77 ng/dL; p < 0.0001), free testosterone (pre 0.96 ± 0.60 pg/mL vs post 0.56 ± 0.30 pg/mL; p < 0.0009), percentage of free testosterone (pre 2.05 ± 1.33% vs post 2.05 ± 1.33%; p < 0.0033) and DHEAS (pre 2.13 ± 0.26 μg/mL vs post 1.70 ± 0.20 μg/mL; p < 0.0001).

**Fig. 3:**
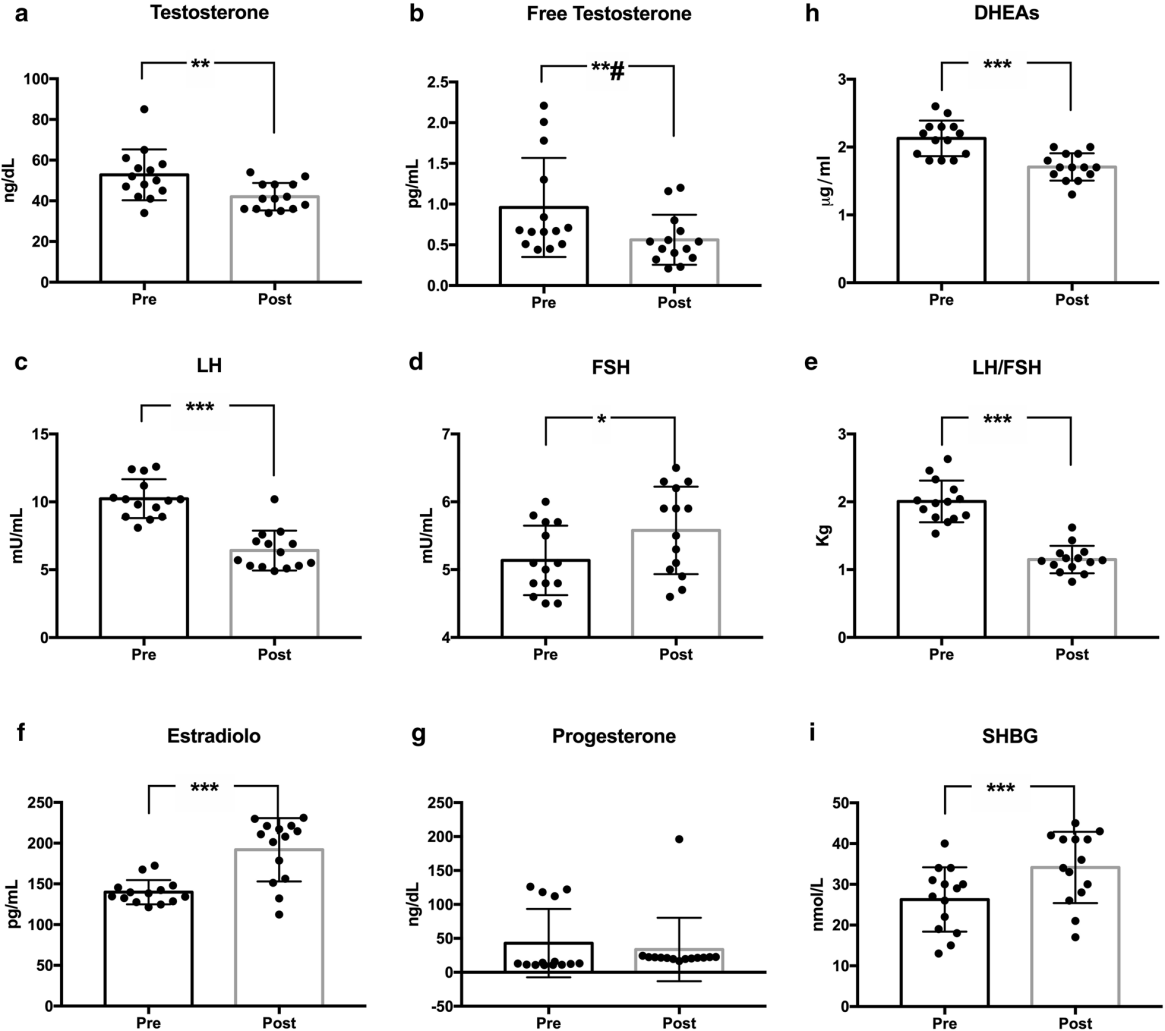
Changes in hormonal variables after 12 weeks of KD. *LH* luteinizing hormone, *FSH* follicle stimulating hormone, *DHEAs* dehydroepiandrosterone sulfate, *SHBG* sex hormone binding globulin. Standard deviation is represented in the figure. ***p < 0.0001; **#p = 0.009; **P = 0.0082; *p = 0.0258

FSH values were found modestly increased, (pre 5.13 ± 0.51 vs post 5.58 ± 0.65; p = 0.0258), in accordance with the finding that usually in PCOS patients the concentration of FSH is not affected by the pathology. Estradiol levels were risen (pre 139.80 ± 14.93 pg/mL vs post 191.90 ± 38.80 pg/mL; p < 0.0001), so did progesterone (pre 12.16 ± 1.41 ng/dL vs post 21.06 ± 1.86 ng/dL; p < 0.0001). SHBG increased significantly from 26.3 ± 7.9 to 34.1 ± 8.7 nmol/L; p < 0.0001 (Fig. 3).

The Ferriman Gallwey Score was slightly reduced, although not significantly (pre 11.36 ± 1.98 vs post 10.57 ± 1.6).

## Discussion

Twelve weeks of LCKD improved almost all anthropometric, biochemical and hormonal variables in a group of 14 women with diagnosed PCOS. PCOS, the most common female endocrinopathy in reproductive age, is characterized by a remarkable phenotypic heterogeneity and should not only be considered a fertility or aesthetic disorder, but a condition associated with a broad spectrum of long-term metabolic and cardio-vascular complications. The causes are not known, but the insulin resistance is considered an important factor etiopathogenetic, that involves 70% of the patients and is in most cases linked to overweight and obesity. Abdominal obesity could be linked to PCOS by a relationship in which it performs the double role of cause and effect: on the one hand, in fact, the increase in visceral fat is favored by hyperandrogenism, on the other it seems to represent an important pathogenetic factor in the development and progression of the PCOS in susceptible women [44].

The Amsterdam ESHRE/ASRM-Sponsored 3rd PCOS Consensus Workshop Group [45] refers that higher BMI is associated with a greater prevalence of menstrual irregularity, hyperandrogenemia and hirsutism. Adipose tissue is, in fact, an extra-glandular source of androgens and its excessive quantity can worsen the hyperandrogenism. Abdominal adipocytes are more active as endocrine cells than adipocytes of the lower portion of the body, which define gynoid obesity: they are more sensitive to catecholamines and less to insulin, with the end result of a hyperinsulinemia compensatory with low-grade inflammation, altered lipid profile, increased production of androgens and low levels of SHBG, which overall favor anovulation. Hyperinsulinemia also influences metabolic flexibility, the ability of skeletal muscle to use alternatively carbohydrates or lipids depending on the availability of energy substrates [46]. Women with PCOS have more reduced metabolic flexibility (evaluated through changes in the respiratory quotient after insulin stimulation) when have hyperandrogenism, high BMI and Insulin resistance [47, 48].

The management of PCOS is essentially symptomatic: when fertility in not the main issue, estroprogestinics represent the choice treatment and are often used for long periods of time. PCOS patients however have an increased cardiovascular risk and a prolonged use of oral contraceptives may be negative effects, so the choice of an estroprogestinic product should be done after a individual cardiovascular risk assessment in all patients. It is not the same to use an estroprogestinic product in a PCOS patient who has normal weight and has normal glucose tolerance and lipid profile or in a PCOS patient who is obese and has metabolic syndrome [49]. The patients, moreover, do not “heal” with the use of the oral contraceptives and after the suspension they often have irregularities of the cycle, so that most of them are forced to undertake therapies to induce ovulation, usually with clomiphene, as suggested the guidelines [12]. Contraception is therefore an acceptable strategy when at the same time an unwanted pregnancy is feared, but for many women a different treatment would be much more advantageous and desirable.

Guidelines [12] propose hormonal contraceptives as first-line management for menstrual abnormalities and hirsutism/acne in PCOS whilst metformin is suggested as treatment in women with PCOS and Type 2 diabetes or impaired glucose tolerance when lifestyle modification fail. For women with menstrual irregularities who have contraindications or intolerance to estroprogestinics, metformin is considered as second-line therapy. According to some experts [50], instead, an attempt with metformin, before administering oral contraceptives, should however be made, regardless of the contraindications, recommending contraception when there is a high risk of unwanted pregnancy. Metformin also seems to improve the subsequent ovarian response to clomiphene in infertility disorders, with comparable results in both very high and non-BMI patients. However, metformin is not free from side effects, which often compromise compliance.

The debate on therapy is therefore still on and there is often confusion, both among the health professionals and among the patients, on who is responsible for the choice of treatment: women usually turn to the gynecologist in the first instance, because have problems of menstrual irregularity and fertility, with disappointment of the endocrinologist. Moreover, many women unwillingly accept the idea of continuous medicalization and seek different solutions.

In this context a central role of a nutritionist could be envisaged, in the light of the indications of the same guidelines, which now believe that a decisive element in the management of PCOS is the modification of lifestyle and nutrition.

Several dietary models have been proposed to correct the metabolic alterations of PCOS, but no one has reached, at the moment, a scientific validation as the best to recommend and it is still not clear even if normal weight, or overweight women may take benefit from a suitable dietary program to improve insulin resistance without caloric restriction. In this context KDs could be considered, as a nutraceutical therapy aimed to increase insulin sensitivity. The data available in the literature [26, 30–32], although few, confirm the assumption that a KD, correcting hyperinsulinemia and improving body composition, can contribute to the normalization of the clinical picture in PCOS. During fasting or a carbohydrate restriction such as a KD, blood insulin concentration decreases, while glucagon increases to maintain the normal blood glucose level, first through glycogen stores, then through the β-oxidation of fatty acids stored in fat depots. Approximately 3–5 days after a very low carbohydrate diet, when the concentration of KBs begins to grow, hunger considerably decreases, but maintaining a state of well-being [51]. The advantage is further substantial if we compare common hypocaloric diets which are strongly restrictive towards the lipids, which keep the level of the orexigenic hormones up to 12 months from the suspension of the diet [52, 53]. In a physiological state of ketosis as during fasting, thanks to the considerable consumption of ketones by the CNS and the balance between insulin and glucagon, ketonemia reaches maximum levels of 7–8 mmol/L [26], with no change in the pH of the blood. During a LCKD the levels of KBs are usually between 0.5–0.6 and 4 [54–56] and a nutritional ketosis could be defined as a blood ketones > 0.5 mmol/L [57–61] Indeed, our subjects showed an steep increase in blood BHB during the first 6 days reaching, at the end of the first week 0.55 ± 0.27 mmol/L BHB, whilst the mean value from day 7 to day 84 was 1.77 ± 0.55 BHB. It is therefore essential to make a clear distinction between physiological ketosis (KD, fasting) and a pathological ketosis, such as that which can occur in diabetes when hyperglycemia and insulin deficiency cause uncontrolled rates of KBs and the ketonemia may exceed 20 mmol/L, exposing to the risk of severe acidosis. Fasting ketosis, however, leads to a loss of protein reserves, especially affecting muscle mass and generating a global state of decay. Conversely, the ketogenic diet, while maintaining a state of ketosis over time due to the limitation of carbohydrates, ensures an adequate supply of protein, preserving the tissues [20]. It is important to underline that a classic KD is not a high-protein diet, but usually has high-fat, adequate-protein, low-carbohydrate content. In fact, an excess of proteins increases gluconeogenesis in the long run, thus affecting the synthesis of KBs: in the first days of a KD the neoglucogenesis from amino acids represents the main source of glucose to keep the glycemia stable, then the demand for amino acids decreases and glucose is synthesized from the glycerol released from adipose tissue by triglyceride hydrolysis. Our diet was a low calorie ketogenic diet in which the amount of protein was high if we consider percentage (32%) but normal if we consider grams of protein per kilogram of body weight (1.23 g pro/Kg bw). Such low calorie approach is more feasible during a KD (LCKD) because it is well known that ketones reduce appetite probably through direct brain actions of KBs [51, 62].

The theoretical assumptions for the use of KD in PCOS are based on the observation that the physiological ketosis induced by a low intake of carbohydrates reduces the levels of circulating insulin and consequently also those of IGF-1, thus suppressing the stimulus on the production of androgens, both ovarian and adrenal. The reduction of circulating lipids, low-grade inflammation and oxidative stress also helps prevent cardiovascular complications [19, 26, 27, 41, 63–65]. Indeed PCOS patients are characterized by higher circulating lymphocytes, monocytes, eosinophilic granulocytes, as well as higher CRP, TNF-α and IL-6, revealing peripheral inflammation. In addition, polycystic ovaries show persistent chronic inflammation with a larger number of infiltrating inflammatory cells. As TNF-a and IL-6 are known to induce insulin resistance, stimulate androgen production and disrupt the hypothalamic-pituitary-ovarian axis, the increased number of lymphocytes could be an factor triggering chronic inflammation and altered hormone secretion [66]. Importantly, hyperglycemia can worsen inflammation and in PCOS patients glucose ingestion induces an inflammatory response that is independent of obesity [67]. Ketogenic diet has demonstrated to be able to improve inflammatory markers per se [41, 68] also due to the action of one of the ketone bodies: 3-hydroxybutyrate [69]. The 14 patients enrolled in this study lost weight with a significant reduction in FBM accompanied by minimal loss of LBM. This result can be related to the inhibition of mTOR by AMPK [70, 71], which is a protective role for muscle anabolism: using KD, in fact, we never expect to get hypertrophy, but to keep the lean mass almost unchanged [72], to difference in extreme caloric restriction diets where the loss is usually more pronounced.

Insulin resistance was significantly reduced, falling below the HOMA-IR threshold of 2.5; also cholesterol and triglycerides were significantly lowered. Androgens decreased significantly, as did LH and LH/FSH, suggesting a regression of the PCOS hormonal anomalies. The improvement in the Ferriman Gallwey Score did not reach statistical significance, however, we can be assumed that 12 weeks were not sufficient to observe a decrease in hirsutism scores: the hair cycle, in fact, depending on the body area can last for some months and it is known that pharmacological therapy based on antiandrogens takes from 6 to 12 months to obtain a good reduction of the score.

## Conclusions

The results of our study are suggestive for a use of the KD as a possible therapeutic aid in PCOS, to be followed by a more balanced dietary regimen, but always with particular attention to the amount of carbohydrates. The duration of KD is still a question: there is no evidence of side effects in the short term, they are considered safe for short cycles. Less information is available on diets in the long term, but the experience gained in the field of epilepsy and GLUT-1 deficiency syndrome [73–79] supports a possible use also for prolonged periods. It is plausible to hypothesize the setting of protocols to be repeated in cycles over time, interspersed with periods of balanced regime.

Some limitations of the study need to be considered. The first is that the effects of a KD on other outcomes as oligomenorrea and infertility were not assessed. Second, an OGTT for glucose and insulin would have added more information about the metabolic effects of a KD, unfortunately the experimental setting didn’t allowed us to perform this analysis; finally, the small sample size and the single arm design therefore further confirmations are required, possibly increasing the number of subjects, extending the treatment period, and match a low calorie ketogenic diet with a low calorie Mediterranean diet.

## Data Availability

The datasets used and/or analysed during the current study are available from the corresponding author on reasonable request.

## Abbreviations

PCOS: Polycystic ovary syndrome
KD: Ketogenic diet
LCKD: Low calorie ketogenic diet
IGF-1: Insulin-like growth factor-1
AMPK: Adenosine 5′-monophosphate-activated protein kinase
HOMA-IR: Homeostasis model assessment–insulin-resistance
mTOR: Mammalian target of rapamycin
IGFBP-1: Insulin-like growth factor binding protein 1
